# Sex determination using circulating cell-free fetal DNA in small volume of maternal plasma in elephants

**DOI:** 10.1038/s41598-019-51641-8

**Published:** 2019-10-24

**Authors:** Boglárka Vincze, András Gáspárdy, Alexandra Biácsi, Endre Ákos Papp, László Garamvölgyi, Endre Sós, Sándor Cseh, Gábor Kovács, Zsolt Pádár, Petra Zenke

**Affiliations:** 1 Department of Animal Breeding and Genetics, University of Veterinary Medicine Budapest, 1078, István utca 2., Budapest, Hungary; 2 Sóstó Zoo, Sóstófürdő, 4431 Sóstói út Nyíregyháza, Hungary; 3 Budapest Zoo and Botanic Garden, 1146 Állatkerti krt. 6-12., Budapest, Hungary; 4 Department of Reproduction and Obstetrics, University of Veterinary Medicine Budapest, 1078, István utca 2., Budapest, Hungary; 5 Research Centre for Forensic Sciences and Criminology, University Széchenyi István, Győr, 9026, Egyetem tér 1., Győr, Hungary; 6 Department of Forensic Medicine, Medical School, University of Pécs, 7624, Szigeti út 12., Pécs, Hungary

**Keywords:** Clinical genetics, Animal breeding

## Abstract

The genetic sexing of animals having long gestation periods offers significant benefits in regard to breeding management among their populations living in captivity. In our study, a new increased-sensitivity PCR method for fetal sexing was developed and tested successfully on elephants, from only a small volume of maternal plasma. Suitable sensitivity was obtained by using short, reduced amplicon lengths with fluorescent labelling for capillary electrophoresis detection. The fundamental principle for this technique was based on the detection of two Y-specific markers (*AmelY* and *SRY*), the presence of which indicates the mother is carrying a male fetus and the absence of these markers designates a female fetus. As a reaction control, the X-chromosomal marker (*PlpX*) was used. To the best of our knowledge, this is the first report on this topic, confirming the presence of fetal cell-free DNA from the plasma of a pregnant captive elephant, and demonstrating a new opportunity for non-invasive assessment in fetal sex determination.

## Introduction

The early prenatal determination of gender through non-invasive diagnosis by PCR amplification of sex-specific or homologous markers is becoming a common method for domesticated as well as for wild animals in captivity. Several tests and PCR‐based methods have been developed for more than a hundred mammalian species^1,2^. Although a significant effect on sensitivity among the most-commonly-used molecular testing techniques has not been revealed, the specificity of real-time quantitative PCR performance could putatively be slightly higher, than conventional PCR^3^. When a DNA sample is in low quantity or degraded, markers with large amplicon sizes prove to be challenging tasks for conventional amplification methods. Peak-high imbalances as well as locus or allele dropouts are both observable in capillary electropherograms, while longer primers allow to increase specificity and to reduce the length of the template necessary for amplification in fragmented DNA samples^4^.

Multiplex PCR of two Y-specific fragments and one X-specific fragment provides a twofold test for male-specific amplification and internal positive control. Species specific primers minimize the risk of cross-amplification in other species, and small amplicon sizes facilitate sensitive analysis of DNA material which was non-invasively obtained^5^.

Approximately 94–97% of cell-free DNA (cfDNA) in pregnant women are of maternal origin^6^ and the majority of these fragments are under 200 base pair (bp) in size^7^ with an even greater portion of them being sub-100 bp nuclear-genomic cfDNA^8^. An increased relative abundance of circular cell-free fetal DNA (ccffDNA) of this ultra-short (<100 bp) size category may be assumed due to the fact that free DNA fragments are not bound to any other molecules, consequently, they have very short half-times (t = 16 min)^6^. Genetic studies have showed a measurable amount of fetal cell-free DNA detectable in maternal blood, however, the sensitivity of DNA-based sex determination does vary according to gestational age, on the volume of maternal blood obtained or copy number of target sequences^9^, in addition to varying among species of domestic animals^10^.

Molecular sexing methods in elephants using short amplicons (<200 bp) also have a substantial relevance in the areas of population ecology, conservation genetics^11,12^ as well as in wildlife forensics^13^. Early and reliable diagnosis of sex in the fetuses of domestic animals has wide-spread and significant commercial and research applications in the livestock industry^10,14,15^, but there is similarly an increasing need for the early determination of fetal sex during pregnancy in zoo species using non-invasive methods and comparable genetic techniques^16^. Ultrasonographic fetal sexing techniques are of limited practical use in larger zoo mammals, particularly in elephants, as the fetus is lying between the huge-sized internal organs of the cow/dam during gestation and is not reachable with ultrasound scanning^17^.

Elephants also display a difference in hormone patterns during pregnancy compared to domestic species and to other elephant genera. Progestagen hormone concentrations are elevated in African elephants during the first half of gestation but then decline to lower levels later than for those measured in Asian elephants, which show a more biphasic pattern^18^. Due to the importance of diagnosis of fetal gender as early as possible as a key for successful breeding management in captive elephant species, there have been studies that showed a fetal gender effect on progestagen concentrations regarding the possible differences in hormone levels in Asian elephants carrying male and female calves^18^.

It was also revealed, that elephant cows carrying bull calves produce more progestagens in utero. An accurate fetal-sexing method has been described previously^19^ based on maternal testosterone levels showing higher concentrations in cases of a male fetus. This testosterone-based method produces a nearly 100% level of accuracy after a year of gestation in Asian elephants but has been reported to be less accurate in African subspecies^19^. Despite this assessment, in Hungary, this method has been implemented successfully in African species: 2/2 male and female carrying pregnancies were screened and produced matching results in both cases when determining the sex of the calves.

In the case of elephants, this new method of genetic-based fetal sexing had not been previously developed. In this study, we investigated the presence of ccffDNA in the maternal plasma of African elephants by developing and using a multiplex PCR system combined with a high-sensitivity capillary electrophoresis for detection. We selected the Y chromosome-specific amelogenin gene (*AmelY*) and the sex-determining region (*SRY*) to detect the male sex of the fetus. Here we report the data from analysis of fetal cfDNA and provide an evaluation of our detection system in regard to accuracy, specificity and sensitivity.

## Methods and Materials

### Sample collection and preparation

All procedures and sampling protocols (blood and hair collection) have been reviewed and approved by the Institutional Animal Care and Use Committee at the Sóstó Zoo and Budapest Zoo & Botanical Garden. Sampling and detection protocols have been reviewed by the Scientific Committee of the University of Veterinary Medicine Budapest (study number 17896-4/2018). All procedures were performed in accordance with relevant guidelines and regulations in force.

For the initial validation of PCR tests, male and female hair samples with intact roots were collected from Asian elephants (*Elephas maximus*) and from African elephants (*Loxodonta africana*) through the contribution of the Budapest Zoo & Botanical Garden and the Sóstó Zoo (Hungary). Hair samples were collected during protected contact training.

Altogether, four blood samples from a male-bearing pregnant elephant (at 80 and 82 weeks of gestation) and from a female-bearing pregnant elephant (at 85 and 86 weeks of gestation, respectively) were collected during anaesthetic procedures associated with routine veterinary examinations. The *vena saphena medialis* was utilised for sampling as described by Mikota^20^. Peripheral blood was collected into gel-clotting activator tubes (Vacutainer® Serum Tube, BD Medical, USA) using an 18 Gauge vacuum-needle unit (Vacutainer^®^ Needle, BD Medical, USA). The skin of the venipuncture-area was cleaned with ethanol immediately prior to sampling.

Blood samples were subsequently centrifuged within 30 minutes of sampling at 1000 × g for approx. 15 minutes. Plasma samples were stored in Eppendorf tubes at −20 °C in small aliquots (approx. 600–750 μL) until the time of the DNA extraction procedure to avoid loss of bioactivity and contamination. There were no repeated freezing/thawing cycles.

### DNA extraction

For the positive and negative controls, genomic DNA was isolated from the approximately 1cm-long root ends of hair samples using a DNeasy^®^Blood &Tissue Kit (Qiagen GmbH, Hilden, Germany), following the procedural guidelines as instructed, resulting in a final volume of 50 μL. Extracted DNA quality was measured on agarose gel using a GelRed^TM^ Nucleic Acid Gel Stain (Biotium, Inc., Izinta Kereskedelmi Ltd., Budapest, Hungary), and concentration was measured by a Qubit 2.0 Fluorometer (Life Technologies Corporation, Biocenter Ltd., Szeged, Hungary), utilizing target-selective dyes which emit fluorescence when bound to DNA.

The four plasma samples were purified and concentrated using a modified “organic/dialysis” method^21,22^. A total of 600 μL of plasma from each sample and a TE buffer of equal volume were combined with 60 μL of proteinase K solution (PCR grade, 20 mg/mL, ThermoFisher Scientific, Bioscience Ltd, Budapest, Hungary) in a 2 mL Eppendorf tube and digested overnight in a 56 °C thermo block. The following day, 780 μL of Ultrapure^TM^ phenol:chloroform:isoamyl alcohol (ThermoFisher Scientific, Bioscience Ltd, Budapest, Hungary) was added to the digested solution. After vortexing (30 sec.) and centrifugation (10 min. at 13.000 rpm), the supernatant was transferred into a sterile 2 mL Eppendorf tube and the previous extraction process was repeated. Microcon^®^-30 centrifugal filter units (Merck Millipore, Merck Ltd., Budapest, Hungary) were used for purification and concentration of the extracted serum solution. Purified DNA was then recovered in 50 μL of TE buffer and concentration was measured by a Qubit 2.0 Fluorometer (Life Technologies Corporation, Biocenter Ltd., Szeged, Hungary).

Extracted DNA from the hair and plasma samples were stored at 4 °C until subsequent analysis.

### Marker selection and primer design

The Y-chromosomal *AmelY* and *SRY* markers were selected for detecting the male sex, and the X-specific proteolipid protein marker (*PlpX*) was applied as an internal positive control. Sequence data from African (GenBank Acc. No.: AY823320, KP141784, AB362890) and Asian elephants (GenBank Acc. No.: AY823325, AF180946, AY823386) were used to design primers for shortened amplicons (Primer Designerv.4.0 software)^23^, resulting in fragments of 75 bp, 85 bp and 147 bp in size, specific to *AmelY*, *SRY* and *PlpX* genes. Limiting the number of primers in the multiplex reaction, the reverse primer for the *SRY* marker was designed to have partial-sequence homology to Asian elephants (Table 1).

**Table 1:** Details of the sex-chromosome specific-markers used.

Marker	Primer sequences 5′-3′ (fw/rev)	5′dye	Anneal T (°C)	Primers (μM)	Size (bp)	GenBank Acc. No.
AmelY	TTCCAGGCAAGGCTAGAACA	6-Fam	47	1	75	MK645854
	ACAACTCAGGGAGGTTTTACG					MK645855
SRY	AGCAAGCTGCTGGGATACCAGTG	6-Fam	53	0.5	85	MK645856
	TATAGTCCGGGTTCTGCGCCTCC					MK645857
PlpX	CTAGCACTGGGTTTGGTTTG	6-Fam	50	0.5	147	MK654898
	CCATATCTGCCTCCCTAGAC					MK654899

### Specificity of the primers

#### Amplification and detection

PCR conditions were optimized as follows, using female and male genomic DNA obtained from the hair of both African and Asian elephants as a positive control. Negative (no-template) reaction controls were applied in each step of analysis to avoid/detect the risk of contamination.

Amplification of *AmelY*, *SRY* and *PlpX* gene fragments were performed in triplex reactions in a 20 μL reaction volume, containing 4 μL DreamTaq™ Green DNA Polymerase (ThermoFisher Scientific, Bioscience, Budapest, Hungary), 2 μL of primer-mix, 2 ng of DNA template and PCR grade H_2_O to volume. Polymerase chain reaction (PCR) was carried out on 2400 Thermal Cyclers (Applied Biosystems, Life Technologies Corp. Co., Budapest, Hungary) using the following conditions: an initial step at 95 °C for 30 sec. followed by 12 cycles of 30 s at 94 °C, annealing of 30 s starting at 51 °C and decreasing by 0.25 °C per cycle and 30 s at 72 °C, followed by 22 cycles of 30 s each at 95 °C, 30 s at 48 °C, 30 s at 72 °C and final extension for 30 min. at 72 °C.

PCR products were separated and analysed by capillary electrophoresis on an ABI Prism 3130XL Genetic Analyzer using GeneScan^TM^-500 LIZ^TM^ Size Standard (ThermoFisher Scientific, Bioscience, Budapest, Hungary). The minimum detection threshold was set at 50 relative fluorescence unit (RFU) during fragment analysis using GeneMapper^®^ ID-X software version 1.4. Bins were set for allele designation for each investigated marker based on allele sizing.

#### Sequencing analysis

*AmelY*, *SRY* and *PlpX* gene fragments were amplified in singleplex PCR (25 μL in volume) containing 5 μL DreamTaq™ Green DNA Polymerase (ThermoFisher Scientific, Bioscience, Budapest, Hungary), 0.5 μM unlabelled forward and reverse primers, 10 ng DNA template and PCR grade H_2_O to volume, under the conditions as delineated above, for subsequent sequencing reactions. PCR amplicons in cases of *PlpX* and *SRY* were purified using GenElute^TM^ PCR Clean-Up Kit (Sigma-Aldrich Co., St. Louis, USA). The size of produced *AmelY* PCR was too short for the GenElute^TM^ purification, therefore the Microcon^®^-100 centrifugal filter units (Merck Kft., Budapest, Hungary) were used instead - in order to retain the DNA and TE buffer to wash out any other confounding factors. For the purpose of revealing overlapping nucleotide sequences, both strands were sequenced two times using the BigDye^®^ Terminator v.1.1 Cycle Sequencing Kit (ThermoFisher Scientific, Bioscience, Budapest, Hungary) in the manner recommended by the manufacturer. For sequence detection, an ABI Prism 3130XL Genetic Analyzer (Applied Biosystems, Life Technologies Corp. Co., Budapest, Hungary) was applied, according to manufacturer’s guidelines. Sequence analysis were performed using Sequencing Analysis Software 5.1 (Applied Biosystems, Life Technologies Corp. Co., Budapest, Hungary), a homology search of resulted sequences was accomplished using the GenBank (BLAST^®^, Basic Local Alignment Search Tool)^24^.

### Sensitivity test of molecular sex determination

In order to estimate the sensitivity of the male-specific analysis method, a mixture of 1 ng/μL of female DNA was used containing 5%, 3%, 2%, 1% and 0.5% male DNA in separate tubes. PCR amplification of 10 μL of the mixture sample was performed, followed by capillary electrophoresis using the conditions described in Section 2.4.1.

### Detection of circulating fetal cfDNA in maternal plasma

PCR amplification of 10 μL of the isolated DNA from maternal plasma was performed, followed by capillary electrophoresis using the conditions described in Section 2.4.1. Fetal sex was confirmed after calving.

## Results and Discussion

### Specificity of the primers

**Figure 1:**
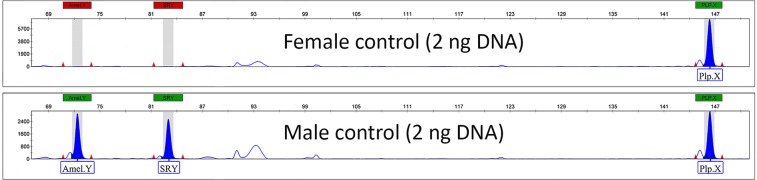
Capillary electropherograms using the triplex PCR method from the female and male control samples (DNA originating from both African and Asian elephants produced equal results).

The specific fragments in question were successfully amplified and detected from positive control samples. The 75 bp, 85 bp and 147 bp amplicons specific to *AmelY*, *SRY* and *PlpX* locus were produced as expected by designed primers in both African and Asian elephant species (Fig. 1).

PCR products were confirmed by sequencing. The sequences were submitted into GenBank (Table 1), which indicated homology with the *AmelY*, *SRY* and *PlpX* genes of African and Asian elephants, with an exception given with the 5′-end region of partially-specific primer in the *SRY* locus of the Asian elephant. This partial homology did not influence the specificity of the result however, as primers do not need to be fully complementary in the 5′ region^4^.

No specific products of *AmelY* and *SRY* genes were detected from the female DNA samples. Although some aspecific fragments having relative low intensity were detected, these artefacts did not influence the reliability of the evaluation of results. Double testing of Y-chromosome loci provided a result of higher accuracy, thus decreasing the chance of a false-positive or false-negative result.

### Sensitivity of molecular sex determination

**Figure 2:**
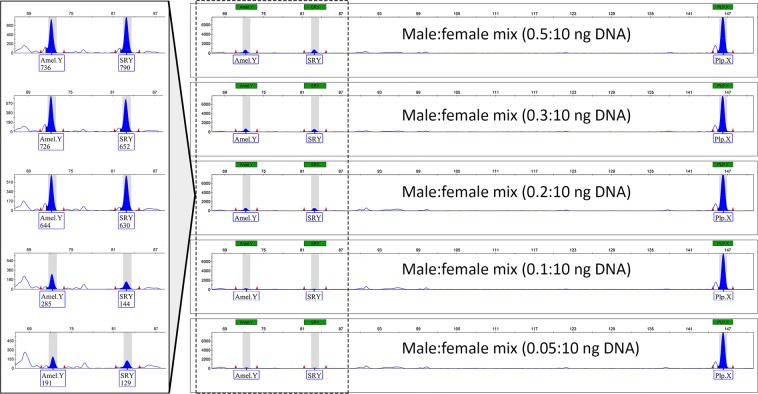
Capillary electropherograms using the triplex PCR method in a male-specific sensitivity test. In the magnified region (on the left side) the number below the marker name indicates the peak height in RFU.

Specific signals on the electropherogram were also detected from the serially-diluted male DNA samples which were intermixed with a huge amount of female DNA. The male-specific fragments at the *AmelY* and *SRY* loci were successfully amplified from the mixtures up to 0.05:10 (50 pg male and 10 ng female DNA) in ratio (Fig. 2), and no Y-chromosomal signals were detectable using only female DNA as a negative control for *AmelY* and *SRY* primers (the electropherogram for this female produced an identical result to the female control in Fig. 1, and therefore is not shown here).

Although the artefact peaks are known to produce signals in close proximity to the specific fragments with less than 2% male-female DNA ratio, due to the specific fragment lengths, both Y-specific markers were able to be clearly identified (Fig. 2).

### Detection of fetal cfDNA in maternal plasma

In investigations of ccffDNA, the extraction method used plays a crucial role, therefore a highly-effective protocol with an increased digestion capacity was used for the purpose of avoiding protein inhibition. Concentration of total ccffDNA was 1–1.2 ng/μL for each plasma sample investigated, which was able to be increased by a larger initial volume of maternal plasma^16,25^. Following PCR amplification with fluorescently-labelled primers, an accurate and highly-sensitive capillary electrophoresis method was performed to detect male and female-specific amplicons. This technique helps to prevent false results and overcome the various drawbacks of traditional identification methods, such as using high cycle numbers (40<) and the reamplification of PCR products (risk of contamination and artefact peaks), and the agarose gel detection (e.g. lower sensitivity, false fragment size).

**Figure 3:**
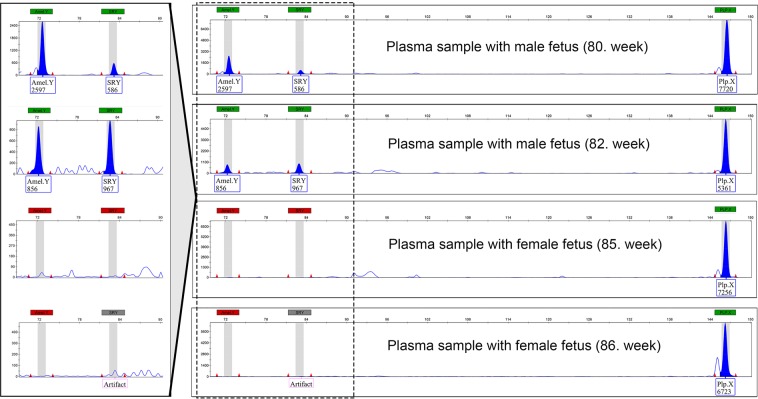
Capillary electropherograms using the triplex PCR method from the four plasma samples obtained from pregnant African elephants.

All four plasma samples produced the female-specific *PlpX* gene fragment, as a reaction control. The presence of fetal Y-chromosome was confirmed in both plasma samples collected from elephants bearing male fetuses by their clearly detectable *AmelY* and *SRY* markers. None of the two female-bearing pregnant elephant samples showed specific fragments of male origin in the analysis (Fig. 3).

### Applicability of results presented and limitations

In this study, a new PCR method has been detailed for the diagnosis of (fetal) sex in two captive elephant genera (*Elephas* and *Loxodonta*) with the goal of helping to make intelligent management decisions in harem planning or the social structures of herds. Through the use of this technique, male samples produce specific fragments for *SRY*, *AmelY* and *PlpX* markers as a positive result, but in female samples, only *PlpX* marker-specific amplicons are able to be detected. Although laboratory equipment is needed to perform DNA extraction, in PCR amplification and analysis, only a blood sample is needed to diagnose the sex of the offspring. Although the laboratory results presented here showed 100% sensitivity and specificity, only a limited number of samples were available for the study itself. Our method has proven to be applicable for both genera (*Elephas* and *Loxodonta*) in the final 2 months of pregnancy, but further studies are needed to determine the earliest possible time and stage of gestation, when the amount of fetal DNA in the maternal blood is sufficient for detecting sex with PCR. The sooner fetal sex can be diagnosed, the better management decisions can be made.

## Conclusions

To our knowledge, this is the first reported examination demonstrating the existence of fetal cell-free DNA in the maternal circulation in elephants. However, although fetal DNA leakage has been proven through this work, more studies are needed to determine the earliest age of gestation in which the presence of male specific markers will be detectable. The method presented here could similarly be an aid for wildlife experts for determining fetal sex from maternal plasma in addition to the fetal testosterone detection method. Due to very short Y-chromosomal amplicon lengths (75 and 85 bp) and the male-specific sensitivity, the application from our developed method is capable of producing successful amplification even from <5% fetal DNA samples and those having highly degraded genetic material. This advanced non-invasive technique is quick, accurate and sensitive for the identification of the sex of the fetus from a relatively small volume of maternal blood or plasma, and may be similarly useful in identifying the Y-chromosome from even a minimal amount of degraded biological materials, such as faeces or any other decomposed remnants of elephants.
